# Informing the Development of a Mobile Phone HIV Testing Intervention: Intentions to Use Specific HIV Testing Approaches Among Young Black Transgender Women and Men Who Have Sex With Men

**DOI:** 10.2196/publichealth.7397

**Published:** 2017-07-07

**Authors:** Beryl A Koblin, Vijay Nandi, Sabina Hirshfield, Mary Ann Chiasson, Donald R Hoover, Leo Wilton, DaShawn Usher, Victoria Frye

**Affiliations:** ^1^ Laboratory of Infectious Disease Prevention New York Blood Center New York, NY United States; ^2^ New York Blood Center New York, NY United States; ^3^ Public Health Solutions New York, NY United States; ^4^ Department of Statistics and Biostatistics Institute of Health, Health Care Policy and Aging Research Rutgers, The State University of New Jersey Piscataway, NJ United States; ^5^ Department of Human Development Binghamton University Binghamton, NY United States; ^6^ Faculty of Humanities University of Johannesburg, Auckland Park Johannesburg South Africa; ^7^ Department of Community Health and Social Medicine CUNY School of Medicine City College of New York New York, NY United States

**Keywords:** HIV infections, African American, homosexuality, male, transgender persons, cell phones

## Abstract

**Background:**

Regular human immunodeficiency virus (HIV) testing of persons at risk is critical to HIV prevention. Infrequent HIV testing and late diagnosis of HIV infection have been observed among young black men who have sex with men (MSM) and transwomen (transgender women)—two groups overrepresented in the HIV epidemic.

**Objective:**

The objective of this study was to inform the development of a brief mobile phone intervention to increase HIV testing among young black MSM and transwomen by providing a tailored recommendation of an optimal HIV testing approach. We identified demographic, behavioral, psychosocial, and sociostructural factors associated with intentions to use three specific HIV testing approaches: self-testing, testing at a clinic or other provider, and couples HIV testing and counseling (CHTC).

**Methods:**

Individuals were eligible for a Web-based survey if they were male at birth; were between the ages of 16 and 29 years; self-identified as black, African American, Caribbean black, African black, or multiethnic black; were not known to be HIV-infected; and reported insertive or receptive anal intercourse with a man or transwoman in the last 12 months. Recruitment occurred via banner advertisements placed on a range of social and sexual networking websites and apps in New York City and nationally, and via events attended by young black MSM and transwomen in New York City. Intention to test by each testing method was analyzed using logistic regression with best subset models and stepwise variable selection.

**Results:**

Among 169 participants, intention to use a self-test was positively associated with comfort in testing by a friend or a partner at home (Adjusted odds ratio, AOR, 2.40; 95% CI 1.09-5.30), and stigma or fear as a reason not to test (AOR 8.61; 95% CI 2.50-29.68) and negatively associated with higher social support (AOR 0.48; 95% CI 0.33-0.72) and having health insurance (AOR 0.21; 95% CI 0.09-0.54). Intention to test at a clinic or other provider was positively associated with self-efficacy for HIV testing (AOR 2.87; 95% CI 1.48-5.59) and social support (AOR 1.98; 95% CI 1.34-2.92), and negatively associated with a lifetime history of incarceration (AOR 0.37; 95% CI 0.16-0.89). Intention to test by CHTC was negatively associated with higher educational level (Some college or Associate’s degree vs high school graduate or less [AOR 0.81; 95% CI 0.39-1.70]; Bachelor’s degree or more vs high school graduate or less [AOR 0.28; 95% CI 0.11-0.70]).

**Conclusions:**

Unique factors were associated with intention to test using specific testing approaches. These data will be critical for the development of a tailored intervention that shows promise to increase comfort and experiences with a variety of testing approaches among young black MSM and transwomen.

## Introduction

Men who have sex with men (MSM) comprised the largest proportion (67%) of new human immunodeficiency virus (HIV) diagnoses in the United States in 2014. Black MSM are affected at greatly disproportionate rates, overall and by age, comprising two-thirds of new diagnoses in the age group of 15 to 29 years [1]. Though national HIV surveillance data are unavailable for transgender women (transwomen) [2], multiple studies report high HIV prevalence and incidence rates, with black transwomen disproportionately affected [3-5].

The Centers for Disease Control and Prevention (CDC) recommends that individuals test every 3 to 6 months if they have additional HIV risk factors [6,7], and recent data from National HIV Behavioral Surveillance (NHBS) found that 65% of HIV-negative men report condomless anal sex with male partners in the prior 12 months [8]. NHBS also reported that HIV testing in the prior 12 months increased among young black MSM from 2008 to 2011 [9]. Nevertheless, infrequent HIV testing and late diagnosis of HIV infection continue to be prevalent among young black MSM and transwomen [3,10,11]. Increasing the uptake of testing is critical to identifying young black MSM and transwomen with undiagnosed HIV infection, linking to them to care, and thereby, lowering HIV transmission in this group.

Delayed HIV testing (eg, not testing in the prior 6 months) among young black MSM and transwomen has been found to be associated with behavioral factors such as condomless sex and substance use [12,13] and psychosocial factors such as stigma associated with HIV and testing [13]. Research into peer norms and social support suggests that stronger social support is associated with a lower risk of delayed HIV testing [12,14]. Socio-structural factors have also been found to be important, such as lack of health insurance, cost of tests and visits, lower income, as well as racism and homophobia experienced at clinic visits [15-18].

Several HIV testing approaches are now available, in addition to traditional clinic-, doctor-, or community-based testing. The Orasure’s OraQuick In-Home HIV Test, approved in 2012, is available via the Internet and at local drug stores and uses an oral swab, providing results to the user in 20 minutes [19]. In addition, couples HIV testing and counseling (CHTC) [20-22] is becoming more prevalent as an important approach, with the given data suggesting that a significant proportion of transmissions among MSM may be attributed to sex with main partners [23,24].

Targeted, tailored, and culturally appropriate HIV testing interventions for young black MSM and transwomen are urgently needed. A number of Web-based and text messaging HIV prevention interventions have been developed for adolescent and young adult MSM [25-31] and a few have targeted uptake of HIV testing as an outcome [30-33]. One intervention, *Get Connected!*, demonstrated an increase in HIV testing through the use of a tailored intervention based on a baseline assessment [30]. Only a limited number of these interventions have been developed specifically for young black MSM or transwomen [27,28].

The overall goal of our study was to develop a brief mobile phone intervention to increase HIV testing by providing young black MSM and transwomen with a tailored recommendation of their optimal HIV testing approach. The development of our intervention is based on theory and results from a multistage process, including formative research [14], the survey reported in this study, community and focus group input, and a pilot study. The formative research and survey were framed broadly within social cognitive theory [34,35] assessing relevant determinants of testing behavior at the personal, behavioral, and socio-structural levels. Within this broad framework, we nested several mid-range theories, including the theory of planned behavior [36], stigma theory [37], social identity theory [38] and social norms theory [35]. These guided our qualitative and quantitative inquiries, resulting in assessments of cognitions; beliefs; behavioral intentions, attitudes, and perceived behavioral control; internalized HIV stigma; personal identity and sense of community; and subjective norms.

In this study, we report on a comprehensive Web-based assessment of demographic, behavioral, psychosocial, and sociostructural factors associated with intentions to use three specific HIV testing approaches (self-testing, testing at a clinic or other provider, and CHTC). The factors found to be associated with HIV testing by specific approaches will be used to construct the HIV testing algorithm, which would provide a tailored recommendation of a person’s optimal testing approach.

## Methods

### Recruitment

Individuals were eligible if they were male at birth; were between the ages of 16 and 29 years; self-identified as black, African American, Caribbean black, African black, or multiethnic black; were not known to be HIV-infected (including those who had never tested for HIV); reported insertive or receptive anal intercourse with a man or transwoman in the last 12 months; and resided in the New York City metropolitan area (for national component described below: resided in the United States). Individuals were ineligible if they were enrolled in any other research study involving HIV testing and/or participating in an HIV vaccine trial. Recruitment for this convenience sample occurred via banner advertisements placed on a range of social and sexual networking websites and apps and by recruitment at local New York City events attended by young black MSM and transwomen. A wide range of images and texts were used to engage potential participants (Multimedia Appendix 1). Persons who clicked on a study banner ad were transferred to the study survey that contained a brief eligibility assessment. Persons who did not meet inclusion criteria were skipped to an exit page, thanked for their interest, and provided a link to locate local HIV testing places. Eligible participants were sent to the Web-based consent form that required acknowledgment of having been read by clicking a Consent button. The consent form presented the purpose of the survey, estimated time needed to complete the survey, and type of questions asked. It also provided assurances of confidentiality and secure data storage, and the name of the principal investigator. Participants were then sent to the Web-based survey, hosted on Health Insurance Portability and Accountability Act–compliant, password- protected servers at Survey Gizmo. At the end of the survey, first name, mobile phone number, and email were collected to facilitate distribution of gift codes for the incentive. Internet Protocol (IP) addresses were collected only for the purpose of prohibiting more than one survey per IP address.

The survey was administered from October 2014 to August 2015 for the New York City metropolitan area initially, with no compensation and then, with a US $10 gift code for survey completion. From June 2015 to July 2015, surveys were completed at New York City events with a US $10 gift card for survey completion. Due to the need to increase the number of responses, we opened the survey nationally for approximately 1 month, from July 2015 to August 2015 with no gift code. Only 7 respondents in the national survey resided in the State of New York. The study was approved by the Institutional Review Boards of the New York Blood Center, Public Health Solutions, and the Binghamton University.

### Measures

The selection of assessment questions was based on our theoretical framework, the literature [39-41] and in-depth interviews with the target populations [14]. Specifically, we report on associations of sociostructural factors, HIV risk factors (sexual risk and substance use), peer norms and social support, and stigma with intention to test by each HIV testing approach. In addition, we describe associations of HIV testing variables such as awareness of testing approaches, comfort levels with different approaches, reasons for not testing, HIV testing self-efficacy, and access to testing with intention to test by each HIV testing approach.

The survey was tested for usability, correct skip patterns, and other functionality prior to launching. The number of items per page and the number of screens varied with participants due to skip patterns. All survey items were required and had an “I would prefer not to answer” response option. Participants were not allowed to review and change answers via a Back button.

#### Outcomes

Three outcomes related to intention to test were defined. Intention to test by a self-test was asked by “In the next 6 months, how likely are you to test using a home HIV test?” with responses of very likely, somewhat likely, somewhat unlikely, and very unlikely. Intention to test at a clinic or other provider was asked by “In next 6 months, how likely are you to test *by yourself* at a clinic, doctor’s office, community-based organization or mobile van?” with the same response categories. Finally, intention to test by CHTC was asked by “In the next 6 months, how likely are you to test *with a partner* at a clinic, doctor’s office, community-based organization or mobile van?” with the same response categories.

#### Sociostructural Variables

Measures of *socioeconomic status* included level of education, employment, and occurrence of financial insecurity: “In the past 3 months, how often was there not enough money in the household for rent, food, or utilities (for example, gas, electric, phone)?” Participants were also asked whether they had *health insurance* and what was their *usual place for medical care*. *Perceived sexual discrimination* was measured by the question: “How often in your life have you been made fun of, picked on, pushed, shoved, hit, or threatened with harm because of being gay, transgender, bisexual or man who had sex with men?” with responses of never, once in a while, sometimes, or a lot. History of *incarceration* was assessed by the question: “Have you ever spent one or more nights in a jail, prison, or detention facility?”

#### HIV Testing Variables

Measures included *awareness* of the self-test and CHTC. The questions about awareness were prefaced with a description of each test: “A home HIV test is one you can buy at a store or online and use to test yourself.” and “Testing Together or Couples Testing is when 2 people talk with a counselor together, get tested together, and get their HIV test results together.” HIV testing self-efficacy was measured with a scale of 7 items such as “I feel confident I could test myself using the home HIV test kit” and “I feel confident I could ask a doctor or health-care provider for HIV testing” and a 4-point Likert response scale from strongly disagree to strongly agree (Cronbach alpha=.81) [42]. *Comfort* with specific testing approaches was asked with 6 different questions such as “How comfortable are you being tested at a clinic outside my community?” with a 3-point Likert response scale of not comfortable, somewhat comfortable, and comfortable [13]. Individuals who had never tested or had not tested in the last year were asked to indicate which of the 11 *reasons they had not tested* such as “I think I’m at low risk for HIV” or “I did not want other people to know that I got a test,” adapted from the CDC’s NHBS System [43]. The reasons were grouped into four categories of reasons for not testing: low risk, stigma or fear, lack of access to testing, and beliefs about treatment/other reason. *Access to testing* was measured with the item “I know where I can get an HIV test” with a 4-point Likert response scale of strongly disagree to strongly agree [44].

#### HIV Risk Behaviors

Questions about *sexual behaviors* in the prior 3 months included number of anal or vaginal sex partners, having a primary partner (“someone who is your boyfriend, girlfriend, lover, life partner, who you live with or see a lot or to whom you feel special emotional commitment”), insertive and receptive anal sex, condom use, and HIV status of partners. Questions on use of *substances* in the prior 3 months included marijuana and stimulants (powder cocaine, crack cocaine, methamphetamine). Occurrence of a *sexually transmitted infection* (STI) in the past year was asked as well. *Risk perception* was measured by 2 questions: “How likely do you think you are to get (HIV/a sexually transmitted disease [STD] other than HIV) in the next year?” with 4-point responses ranging from very unlikely to very likely.

#### Peer Norms, Social Support and Stigma

*Peer norms for HIV testing* were asked using two questions: “Most of my friends would approve of me getting an HIV test” and “My friends would probably think less of me if they knew I got an HIV test” with a 4-point Likert response scale of strongly disagree to strongly agree [45]. *Social support related to HIV and sex* was asked with a scale of 3 items, including “How often do you have someone to share concerns about HIV/AIDS” with a 4-point Likert response scale of never to all the time (Cronbach alpha=.92) [46]. *HIV stigma* was measured with a scale of 8 items such as “If you talk too much about HIV, people will think that you have HIV” with a 4-point response scale of strongly disagree to strongly agree (Cronbach alpha=.88).

### Statistical Analysis

Consistent with best practices in Web-based survey research [47] and due to the high eligibility rate in the New York City survey with compensation, a systematic review was made of all completed surveys to identify potentially invalid survey entries by identifying surveys with the following characteristics and patterns among multiple records: (1) survey completion in less than 10 minutes; (2) female first name (and not self-identified as a transwoman); (3) IP address outside of the United States; (4) email address that was similar to others or made up of many consonants (eg, xyzztp); (5) mobile phone numbers with same first 7 digits (eg, 212-568-4xxx); (6) IP addresses that matched on 3 of the 4 quadrants; and (7) repeated identical answers. Through this process, we identified 251 invalid responses in the New York City metropolitan area survey, which had compensation. No invalid surveys were identified in the national survey, which did not have compensation.

A comparison was made between participants from the local survey to those from the national survey. Those from the national survey who indicated that they resided in the New York City metro area were classified as local survey respondents. Age group, ethnicity, educational level, financial insecurity, living situation, being a transwoman, and intent to test in the next 6 months by self-test, by clinic/other provider, and by CHTC were all not significantly associated with national/local survey status (data not shown). Only one demographic variable, employment, was found to be significantly associated with national/local survey status, with 60% (36/60) of respondents from the national survey employed full-time compared with 34.6% (37/107) of respondents from the local survey. Given the limited differences between the national and local survey respondents, analyses were conducted on the survey data as a whole.

Survey responses for the outcome measures were recoded into binary variables that maintained similar distributions across the outcomes. As such, intention to test by a self-test and intention to test by CHTC were classified as very likely and somewhat likely versus somewhat unlikely and very unlikely. Intention to test at a clinic or other provider was classified as very likely versus all other categories. In this manner, we had sufficient sample size in each category to conduct analyses to identify factors associated with each outcome.

For survey scales, a mean score of the scale items was generated based on complete cases. Separate bivariate analyses were conducted for each of the three intention-to-test outcomes. Chi-square tests for binary and categorical measures and nonparametric Kruskal-Wallis tests for continuous measures were conducted to test significant associations between intention to test by a specific testing approach and socio-structural variables, HIV testing variables, HIV risk behaviors, peer norms, social support, and stigma at *P*<.05.

The three testing method outcomes then were analyzed individually in two stages. Due to the comprehensive list of HIV risk behaviors and HIV testing feature variables, in the first stage, we implemented logistic regression modeling with best subset models (Best=4) [48] on these measures. In the second stage, we conducted stepwise variable selection [48] adding the peer norms and social support, stigma, and sociostructural factors to the variables identified in the best subset from the first stage. For the intention to test by CHTC outcome, we forced primary partner status into the final model.

## Results

### Study Sample

**Figure 1:**
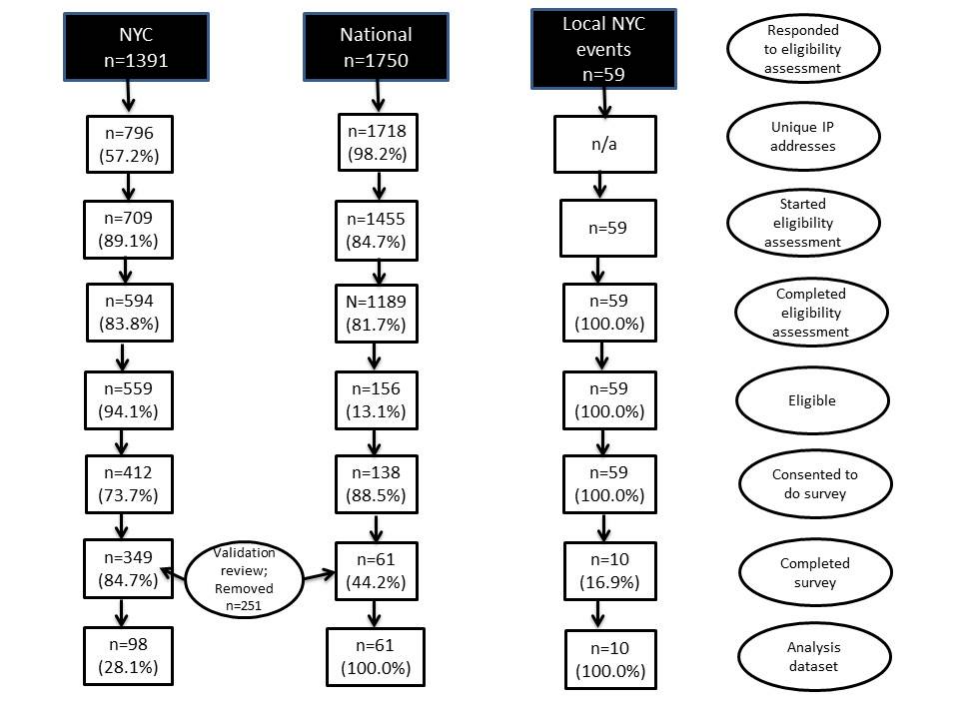
Response rates and analysis dataset.

Figure 1 presents the number of responses through the recruitment process. The final sample was 169 completed surveys: 98 from the New York City survey, 61 from the national survey, and 10 from the local New York City events.

The mean age of respondents was 24.1 years (Standard deviation, SD=3.0); the majority ethnicity was African American (Table 1). About 1 in 7 respondents had never tested for HIV and over one-quarter had their last HIV test over 6 months ago. Awareness of self-testing and CHTC was relatively high, with 70.4% of participants indicating that they knew about self-tests, and 55.0% indicating that they knew about CHTC. However, only 11.1% (16/144) of those who previously tested had ever used an HIV self-test and 13.2% (19/144) had ever tested using CHTC. Almost all (133/140; 95.0%) tested at a clinic for their last test.

**Table 1:** Demographic characteristics and intention to test, All About Me Study, N=169.

Characteristics		n (%)
**Age, years**		
	16-19	12 (7.1)
	20-24	82 (48.5)
	25-29	75 (44.4)
**Ethnicity**		
	African American	137 (81.1)
	Caribbean	34 (20.1)
	Afro-Latino	17 (10.1)
	African	3 (1.8)
	Other	7 (4.1)
Transwoman		14 (8.3)
**Recent HIV testing**		
	In last 3 months	57 (33.9)
	4-6 months ago	41 (24.4)
	7-12 months ago	25 (14.9)
	More than 1 year ago	21 (12.5)
	Never	24 (14.3)
**Region of survey respondents^a^**		
	New York City	108 (63.9)
	South	33 (19.5)
	Northeast	12 (7.1)
	Midwest	12 (7.1)
	West	4 (2.4)

^a^based on US Census Bureau regional divisions.

**Figure 2:**
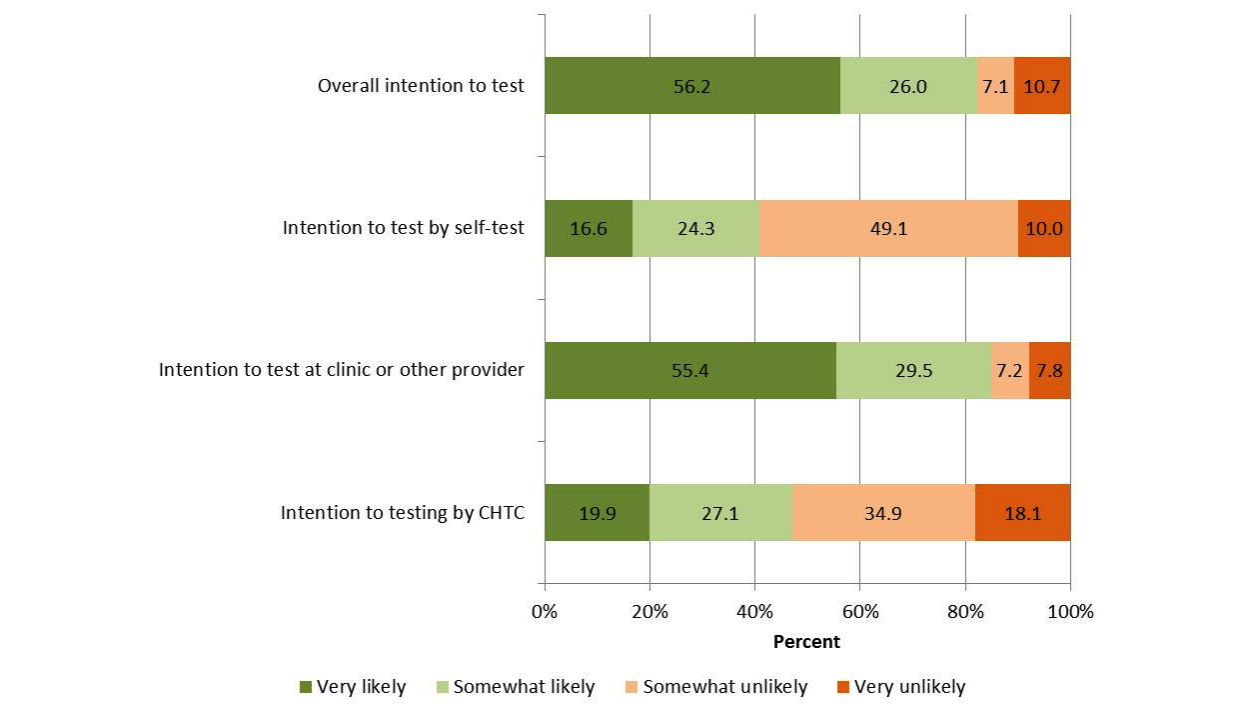
Intention to test by specific testing approaches.

With regard to specific testing approaches, 40.9% and 47.0% were very or somewhat likely to test in the next 6 months by self-test and CHTC, respectively (Figure 2). Over half (55.4%) of participants were very likely to test in the next 6 months at a clinic or other provider. Participants who had not tested in the last 6 months were significantly more likely to be very likely or somewhat likely to test by self-test in the next 6 months (40/70; 57.1%) compared with participants who had tested in the prior 6 months (29/98; 29.6%; *P*=.001). In contrast, participants who had not tested in the last 6 months were significantly less likely to be very likely to test at a clinic or other provider in the next 6 months (28/67; 41.8%) compared with those who had tested recently (63/98; 64.3%; *P*=.007). No significant differences in intention to test by CHTC were observed by recent testing.

### Correlates of Intention to Test by Specific Testing Approaches: Sociostructural Variables

Intention to test by self-test was significantly higher among those without health insurance and for those whose usual place of care was a community health center/clinic (Table 2). Intention to test at a clinic or other provider was significantly lower for participants with a lifetime history of incarceration. Intention to test by CHTC was significantly higher with lower educational levels.

**Table 2:** Correlates of intention to test by specific testing approaches: Sociostructural variables, All About Me Study, N=169.

Sociostructural variables		Intention to test by...					
		Self-test^a^		Clinic or other provider^b^		CHTC^a,c^	
		%	*P*	%	*P*	%	*P*
**Education**			.13		.43		.008
	High school graduate or less, vocational	20 (40.8)		24 (51.1)		27 (56.3)	
	Some college	38 (47.5)		42 (53.2)		41 (52.6)	
	College degree or more	11 (28.2)		25 (64.1)		10 (25.6)	
**Financial insecurity**			.14		.19		.93
	Very often	9 (39.1)		12 (54.6)		11 (47.8)	
	Fairly often	14 (56.0)		9 (36.0)		10 (40.0)	
	Once in a while	18 (50.0)		20 (58.8)		16 (48.5)	
	Never	27 (33.3)		49 (60.5)		37 (45.7)	
**Employment**			.48		.91		.11
	Full-time	33 (45.2)		39 (54.2)		38 (53.5)	
	Part-time	18 (42.9)		23 (54.8)		14 (33.3)	
	Off the books/not working/other	18 (34.6)		29 (58.0)		24 (47.1)	
**Health insurance**			.02		.93		.31
	No	20 (58.8)		18 (54.6)		18 (54.6)	
	Yes	48 (36.4)		72 (55.4)		58 (44.6)	
**Usual place for medical care**			.001		.38		.58
	Community health center/clinic	27 (64.3)		19 (46.3)		22 (53.7)	
	Private MD/student health center	22 (28.6)		47 (61.8)		33 (42.9)	
	Emergency room/urgent care	14 (35.9)		21 (55.3)		18 (48.7)	
	Alternative practitioner/nowhere	5 (55.6)		4 (44.4)		3 (33.3)	
**Perceived sexual discrimination (lifetime)**			.15		.77		.10
	Never	22 (38.6)		30 (55.6)		30 (55.6)	
	Once in a while	17 (32.7)		29 (55.8)		26 (50.0)	
	Sometimes	17 (58.6)		14 (48.3)		13 (44.8)	
	A lot	12 (41.4)		18 (62.1)		8 (27.6)	
**Incarceration (lifetime)**			.39		.008		.38
	No	51 (39.5)		78 (60.9)		57 (44.5)	
	Yes	18 (47.4)		13 (36.1)		19 (52.8)	

^a^Self-test and CHTC outcomes: Very likely/somewhat likely versus somewhat unlikely/very unlikely.

^b^Clinic outcome: Very likely versus somewhat likely/somewhat unlikely/very unlikely.

^c^CHTC: couples HIV testing and counseling.

### Correlates of Intention to Test by Specific Testing Approaches: HIV Testing Variables

Intention to test by self-test was significantly higher for participants who were previously unaware of the self-test and among those somewhat comfortable testing at a clinic inside the community (but not comfortable testing outside the community), by their health care provider, on a mobile van, and by a friend or partner at home (Table 3). Participants who indicated stigma/fear or lack of access to testing as reasons not to test were significantly more likely to indicate an intention to test by self-test.

**Table 3:** Correlates of intention to test by specific testing approaches: HIV testing variables, All About Me Study, N=169.

HIV testing variables			Intention to test by...					
			Self-test^a^		Clinic or other provider^b^		CHTC^a,c^	
			%	*P*	%	*P*	%	*P*
**Aware of self-test**				.001		.001		.18
	No		30 (60.0)		17 (34.7)		27 (55.1)	
	Yes		39 (32.8)		75 (64.1)		51 (43.6)	
**Aware of CHCT**				.06		.09		.83
	No		37 (48.7)		35 (48.0)		35 (48.0)	
	Yes		32 (34.4)		57 (61.3)		43 (46.2)	
**Comfort getting tested…**								
	**At clinic in my community**			.009		<.001		.01
		Not comfortable	12 (42.9)		8 (29.6)		7 (25.9)	
		Somewhat comfortable	21 (63.6)		10 (30.3)		21 (63.6)	
		Comfortable	36 (33.6)		74 (69.8)		50 (47.2)	
	**At clinic outside my community**			.04		.002		.55
		Not comfortable	13 (61.9)		5 (23.8)		8 (40.0)	
		Somewhat comfortable	17 (48.6)		17 (48.6)		19 (54.3)	
		Comfortable	39 (34.8)		70 (63.6)		51 (46.0)	
	**By my health care provider**			.004		<.001		.18
		Not comfortable	5 (38.5)		4 (30.8)		4 (30.8)	
		Somewhat comfortable	22 (66.7)		8 (24.2)		19 (59.4)	
		Comfortable	42 (34.7)		79 (66.4)		55 (45.8)	
	**On a mobile van**			.02		.001		.67
		Not comfortable	12 (30.8)		22 (56.4)		16 (41.0)	
		Somewhat comfortable	25 (59.5)		14 (33.3)		21 (50.0)	
		Comfortable	32 (37.7)		56 (67.5)		41 (48.8)	
	**By myself at home**			.13		.43		.45
		Not comfortable	10 (27.0)		23 (62.2)		20 (54.1)	
		Somewhat comfortable	14 (43.8)		15 (46.9)		12 (38.7)	
		Comfortable	45 (45.9)		54 (56.8)		46 (47.9)	
	**By a friend or partner at home**			.007		.22		.05
		Not comfortable	23 (31.1)		44 (59.5)		28 (38.4)	
		Somewhat comfortable	21 (63.6)		14 (42.4)		21 (63.6)	
		Comfortable	25 (43.9)		33 (58.9)		28 (50.0)	
	**With a sex partner**			.26		.009		.001
		Not comfortable	19 (45.2)		17 (40.5)		10 (23.8)	
		Somewhat comfortable	21 (51.2)		20 (48.8)		22 (55.0)	
		Comfortable	29 (36.3)		53 (68.0)		46 (58.2)	
**Reasons for not testing**								
	**Low risk**			.06		.32		.30
		No	58 (38.4)		85 (56.7)		68 (45.6)	
		Yes	11 (61.1)		7 (43.8)		10 (58.8)	
	**Stigma/fear**			<.001		.004		.38
		No	53 (35.8)		87 (59.6)		70 (48.3)	
		Yes	16 (76.2)		5 (25.0)		8 (38.1)	
	**Lack of access to testing**			.04		.01		.03
		No	57 (38.0)		87 (58.8)		74 (50.0)	
		Yes	12 (63.2)		5 (27.8)		4 (22.2)	
	**Belief about treatment/other**			.47		.41		.82
		No	67 (41.6)		90 (56.3)		75 (47.2)	
		Yes	2 (25.0)		2 (33.3)		3 (42.9)	
	**Know where to test**			.79		.03		.12
		Strongly disagree/disagree	5 (35.7)		4 (30.8)		3 (25.0)	
		Strongly agree/agree	57 (39.3)		88 (61.1)		70 (48.3)	

^a^Self-test and CHTC outcomes: Very likely/somewhat likely versus somewhat unlikely/very unlikely.

^b^Clinic outcome: Very likely versus somewhat likely/somewhat unlikely/very unlikely.

^c^CHTC: couples HIV testing and counseling.

Intention to test at a clinic or other provider was significantly higher for those who were aware of the self-test, were comfortable testing at a clinic inside and outside the community, by their health care provider, on a mobile van or with a sex partner, and reported knowing where to test for HIV. Participants who indicated stigma/fear or lack of access to testing as reasons not to test were significantly less likely to indicate an intention to test at a clinic or other provider. The mean score for HIV testing self-efficacy was significantly higher for participants who indicated an intention to test at a clinic or other provider compared with those who did not indicate an intention to test at a clinic or other provider (Table 4).

Participants who were somewhat comfortable testing at a clinic inside the community or somewhat comfortable or comfortable testing with a sex partner had a significantly higher intention to test by CHTC (Table 3). Participants who indicated lack of access to testing were significantly less likely to indicate an intention to test by CHTC compared with those who did not.

### Correlates of Intention to Test by Specific Testing Approaches: HIV Risk Behaviors

Participants with a primary partner and reporting an STI in the prior year were significantly more likely to indicate an intention to test by self-test (Table 5). Marijuana users were significantly less likely to indicate an intention to test by self-test compared with non-users, whereas stimulant users were more likely to indicate an intention to test by self-test compared with non-stimulant users. The mean score for risk perception was significantly higher for participants who indicated an intention to test by self-test compared with those who did not indicate an intention (Table 4).

Stimulant users were less likely to indicate an intention to test at a clinic or other provider compared with non-stimulant users (Table 5). Participants who reported having a primary partner were more likely to indicate an intention to test by CHTC compared with those not in a primary partnership (40.4%), although this was of borderline significance (Table 5).

### Correlates of Intention to Test by Specific Testing Approaches: Peer Norms, Social Support and Stigma

Mean scores for peer norms for testing and social support were significantly lower for those with an intention to test by self-test compared with those who did not (Table 4). Conversely, mean scores for peer norms for testing and social support were significantly higher for those with an intention to test at a clinic or other provider test compared with those who did not. The mean score for peer norms for testing was significantly lower for those with an intention to test by CHTC compared with those who did not.

**Table 4:** Correlates of intention to test by specific testing approaches: HIV testing self-efficacy, risk perception, peer norms, social support and stigma, All About Me Study, N=169.

Self-efficacy, risk perception, norms, support and stigma		Intention to test by...					
		Self-test^a^		Clinic or other provider^b^		CHTC^a,c^	
		Mean (SD)	*P*	Mean (SD)	*P*	Mean (SD)	*P*
**HIV testing self-efficacy**			.45		<.001		.08
	Intention	3.2 (0.6)		3.3 (0.5)		3.2 (0.6)	
	No intention	3.1 (0.7)		2.9 (0.7)		3.0 (0.7)	
**Risk perception**			.002		.14		.79
	Intention	2.2 (0.9)		1.8 (0.9)		2.0 (0.9)	
	No intention	1.7 (0.8)		2.0 (0.9)		1.9 (0.9)	
**Peer norms for testing**			<.001		<.001		.01
	Intention	3.3 (0.7)		3.7 (0.7)		3.4 (0.8)	
	No intention	3.7 (0.6)		3.4 (0.7)		3.7 (0.6)	
**Social support**			<.001		<.001		.93
	Intention	2.4 (0.8)		3.2 (0.9)		2.8 (1.0)	
	No intention	3.0 (1.0)		2.4 (0.9)		2.8 (1.0)	
**HIV stigma**			.09		.09		.83
	Intention	17.9 (5.6)		19.8 (5.7)		19.0 (5.6)	
	No intention	19.7 (6.4)		17.9 (6.2)		18.8 (6.4)	

^a^Self-test and CHTC outcomes: Very likely/somewhat likely versus somewhat unlikely/very unlikely.

^b^Clinic outcome: Very likely versus somewhat likely/somewhat unlikely/very unlikely.

^c^CHTC: couples HIV testing and counseling.

### Multivariable Analysis for Intention to Test by Specific Approaches

In multivariable analysis (Table 6), intention to use a self-test remained independently associated with comfort in testing by a friend or partner at home and stigma or fear as a reason not to test and negatively associated with higher social support and having health insurance. Intention to test at a clinic or other provider remained independently associated with self-efficacy for HIV testing and higher social support and negatively associated with a lifetime history of incarceration. Intention to test by CHTC remained negatively associated with higher educational level and having a primary partner was of borderline significance.

**Table 5:** Correlates of intention to test by specific testing approaches: HIV risk behaviors, All About Me Study, N=169.

HIV risk behaviors in the last 3 months		Intention to test by...					
		Self-test^a^		Clinic or other provider^b^		CHTC^a,c^	
		%	*P*	%	*P*	%	*P*
**Number of anal/vaginal partners**			.47		.96		.24
	0-1	24 (40.0)		32 (55.2)		31 (52.5)	
	2-3	25 (48.1)		30 (58.8)		26 (52.0)	
	4-5	10 (35.7)		15 (53.6)		10 (35.7)	
	>5	8 (30.8)		14 (53.9)		9 (34.6)	
**Primary partner**			.01		.22		.05
	No	33 (32.7)		59 (59.6)		40 (40.4)	
	Yes	35 (52.2)		33 (50.0)		37 (56.1)	
**Condomless anal intercourse (insertive or receptive)**			.18		.09		.16
	No	29 (47.5)		28 (46.7)		33 (54.1)	
	Yes	40 (37.0)		64 (60.4)		45 (42.9)	
**Anal intercourse with positive or unknown status partner**			.16		.78		.56
	No	52 (38.2)		73 (54.9)		64 (48.1)	
	Yes	17 (51.5)		19 (57.6)		(14) 42.4	
**STI^d^in past year**			.04		.33		.31
	No	43 (35.8)		62 (53.0)		52 (44.4)	
	Yes	26 (53.1)		30 (61.2)		26 (53.1)	
**Marijuana use**			.03		.19		.46
	No	39 (49.4)		39 (50.0)		39 (50.0)	
	Yes	30 (33.3)		53 (60.2)		39 (44.3)	
**Stimulant use**			.009		.03		.35
	No	56 (37.3)		86 (58.5)		71 (48.3)	
	Yes	13 (68.4)		6 (31.6)		7 (36.8)	

^a^Self-test and CHTC outcomes: Very likely/somewhat likely versus somewhat unlikely/very unlikely.

^b^Clinic outcome: Very likely versus somewhat likely/somewhat unlikely/very unlikely.

^c^CHTC: couples HIV testing and counseling.

^d^STI: Sexually transmitted infection.

**Table 6:** Multivariable analysis for intention to test by specific testing approaches, All About Me Study, N=169.

Variables	Intention to test by...					
	Self-test^a^		Clinic or other provider^b^		CHTC^a,c^	
	AOR^d^	95% CI	AOR	95% CI	AOR	95% CI
Comfort in testing by a friend or partner at home	2.4	1.1-5.3				
Stigma or fear as a reason not to test	8.6	2.5-29.7				
Social support (per point higher)	0.5	0.3-0.7	2.0	1.3-2.9		
Health insurance	0.2	0.1-0.5				
Self-efficacy for HIV testing (per point higher)			2.9	1.5-5.6		
Lifetime history of incarceration			0.4	0.2-0.9		
Some college/Associate’s degree vs high school graduate or less					0.8	0.4-1.7
Bachelor’s degree or higher vs high school graduate or less					0.3	0.1-0.7
Have a primary partner					1.8	1.0-3.5

^a^Self-test and CHTC outcomes: Very likely/somewhat likely versus somewhat unlikely/very unlikely.

^b^Clinic outcome: Very likely versus somewhat likely/somewhat unlikely/very unlikely.

^c^CHTC: couples HIV testing and counseling.

^d^AOR: adjusted odds ratio.

## Discussion

### Principal Findings

With multiple options available to test for HIV, but low uptake of testing among black MSM and transwomen, data from this study serve as the basis for development of a computerized algorithm that provides a tailored recommendation of an optimal HIV testing approach for individuals. Young MSM and transwomen completed a Web-based comprehensive assessment regarding HIV testing history and related experiences, awareness and comfort levels with specific testing modalities, sociostructural factors, behavioral risk, peer norms, social support and stigma. The main outcomes of the assessment centered on intentions to test using specific HIV testing approaches. We sought to identify correlates of intention to test by three specific HIV testing methods in order to inform the basis of the testing algorithm. Using equations from the multivariable models generated from these analyses, we have calculated the probability of intention to test for each specific testing approach. We developed decision rules for choosing the recommended HIV testing approach. Thus, from a short series of questions, we can provide a tailored recommendation of an optimal testing approach and are currently pilot testing this intervention algorithm among young black MSM and transwomen. One example is a young black MSM with some college education and health insurance. He is not comfortable testing at home with a friend or partner. He has a high level of social support and HIV testing self-efficacy. He does not cite stigma/fear as a reason not to test. Using the algorithm and the decision rules, this individual would receive a recommendation of “Based on your answers, a good option for your next HIV test is going to an HIV testing site, clinic or doctor.” Another example is someone with some college education and health insurance. He has a primary partner. He has a history of incarceration. He is comfortable testing at home with a friend or partner. He is on the lower range of social support but in the medium range of HIV testing self-efficacy. He cites stigma/fear as a reason not to test. This individual would receive a recommendation of “Based on your answers, a good option for your next HIV test is an HIV self-test.”

We found that over half of the participants (55%) were very likely to test at a clinic or other provider in the next 6 months. These results are similar to those found in an national Web-based survey of mostly white and Latino MSM conducted in 2012, with 56% indicating that they would be extremely likely to test at a physician’s office [49]. A small proportion of participants indicated that they were very likely to test by self-test (17%) or CHTC (19%). These are considerably lower than the 58% and 30% found to be extremely likely to test by self-test or CHTC, respectively, in the national Web-based survey [49]. Although a high percentage of participants knew about these approaches, the lower intention to test using these methods may reflect a generalized reluctance to adopt newer approaches [14]. Alternatively, there may be unique aspects of each testing approach that appeal, either by design or otherwise, to select members of the population.

In support of this notion, we found in multivariable analysis that specific variables were associated with intention to test using specific testing approaches. For example, those who cited stigma or fear as a reason not to test were more likely to express an intention to use a self-test. Other studies have found that perceived stigma is negatively associated with recent HIV testing among young MSM [50-52] and among young black MSM [13,53]. The privacy associated with self-testing may address the stigma and fear associated with HIV testing at a clinic site or in front of another person. Our results support the idea that the HIV self-test may be effective in increasing HIV testing uptake among those for whom stigma or fear forms a barrier to HIV testing. Our previous qualitative work with young black MSM and transwomen found that privacy was an important part of the appeal of self-testing, in addition to how self-testing reduced the anxiety of going to a clinic [14]. In our analysis, we also found that having higher social support was negatively associated with intention to test by self-test. Thus, self-testing may provide an option for those who are not well-linked with a supportive social network. Our qualitative work suggested that lower social support could reflect the need for autonomy or control over the testing experience [14].

As expected, we found that a higher level of comfort with testing by a friend or partner at home was also associated with intention to test by self-test. In addition, lack of health insurance was also associated with a higher intention to test by self-test. Others have found structural barriers to testing, such as lack of health insurance, to be higher among black MSM compared with white MSM [54], whereas others have found no difference [55]. Perhaps such individuals are unaware that HIV testing is available for free at many clinics, or health insurance may be a marker for other issues such as inadequate access to health care or having experienced discrimination in health care settings [13,15,56]. Attempts to increase access to self-test kits have been conducted by local health departments such as a recent public health initiative in New York City, which provided a limited number of free self-test kits [57].

Intention to test at a clinic or other provider was more likely with higher HIV testing self-efficacy and social support. We are not aware of other studies among young black MSM and transwomen, which provide data on the role of self-efficacy and social support in intention to test by specific HIV testing approaches. Recent studies examining the role of social network and individual-level characteristics in HIV testing behaviors found that some social network characteristics and functions such as network-mediated information acquisition about HIV/AIDS was associated with ever and repeat testing, but HIV-specific social support from network members was not associated with ever, repeat, or recent HIV testing [52]. We also found that those who had a lifetime history of incarceration were less likely to have an intention to test at a clinic or other provider. It is possible such intentions are low because they were HIV tested while incarcerated; alternatively, they may have had negative experiences testing in the criminal justice system [58,59].

Finally, only lower educational level was found to be associated with intention to test by CHTC. Sharma et al [49] also found that lower educational level was associated with the likelihood to test by CHTC among MSM in a national Web-based survey. Perhaps these findings are explained by partnering patterns and duration of relationships by educational level or whether CHTC is available at community clinics versus private practices.

### Limitations

This study has several limitations. Due to the limited sample size, especially among transwomen, we may not have detected some associations of importance. A large number of invalid cases were detected from the Web-based New York City survey; a dilemma that researchers face when conducting Web-based studies is whether or not to provide monetary incentives, which can help to attract potential participants but also invites opportunities for fraudulent data [60]. In addition, even with fraud detection protocols in place (eg, reCAPTCHA codes, blocking duplicate IP addresses, verifying email and phone numbers) with Web-based recruitment, many participants can enroll during a short time frame, or an individual can attempt to participate numerous times [61], making it difficult to prevent duplicate respondents and invalid data in real time.

Addressing and removing suspicious and invalid cases is critical, as those cases may differ from valid cases, potentially affecting study outcomes and implications [62,63]. Finally, recruitment occurred through a range of social and sexual networking websites and apps and at local events. It is likely that we missed some individuals who do not visit the specific websites or attend local events, or who do not publicly identify as MSM or transgender.

### Conclusions

It is a crucial public health goal to increase the proportion of young black MSM and transwomen who get tested for HIV and test consistently. Multiple websites and apps are rapidly becoming available to maximize user choice to increase levels of intervention uptake, such as contraceptives, pre-exposure prophylaxis, and STI testing (eg, Nurx app, “Which Method,” “I want the Kit”). Given the need to increase regular HIV testing among young black MSM and transwomen, the data presented here provide information on the important factors that are associated with intentions to test using these different approaches. These data will be critical for the development of a tailored intervention that shows promise to increase comfort and experiences with a variety of testing approaches among young black MSM and transwomen.

## Appendix

Multimedia Appendix 1 Sample recruitment ads and sources.

## Abbreviations

AOR: adjusted odds ratio
CDC: Centers for Disease Control and Prevention
CHTC: couples HIV testing and counseling
HIV: human immunodeficiency virus
IP: Internet Protocol
MSM: men who have sex with men
NHBS: National HIV Behavioral Surveillance
SD: standard deviation
STD: sexually transmitted disease
STI: sexually transmitted infection

## References

[1] Centers for Disease Control and Prevention.

[2] (2015). Centers for Disease Control and Prevention.

[3] Habarta N, Wang G, Mulatu MS, Larish N (2015). HIV testing by transgender status at Centers for Disease Control and Prevention-funded sites in the United States, Puerto Rico, and US Virgin Islands, 2009-2011. Am J Public Health.

[4] Herbst JH, Jacobs ED, Finlayson TJ, McKleroy VS, Neumann MS, Crepaz N, HIV/AIDS Prevention Research Synthesis Team (2008). Estimating HIV prevalence and risk behaviors of transgender persons in the United States: a systematic review. AIDS Behav.

[5] Rapues J, Wilson EC, Packer T, Colfax GN, Raymond HF (2013). Correlates of HIV infection among transfemales, San Francisco, 2010: results from a respondent-driven sampling study. Am J Public Health.

[6] Branson BM, Handsfield HH, Lampe MA, Janssen RS, Taylor AW, Lyss SB, Clark JE, Centers for Disease ControlPrevention (CDC) (2006). Revised recommendations for HIV testing of adults, adolescents, and pregnant women in health-care settings. MMWR Recomm Rep.

[7] Workowski KA, Bolan GA, Centers for Disease Control and Prevention (2015). Sexually transmitted diseases treatment guidelines, 2015. MMWR Recomm Rep.

[8] Centers for Disease Control and Prevention (2016). CDC.

[9] Cooley LA, Oster AM, Rose CE, Wejnert C, Le BC, Paz-Bailey G (2014). Increases in HIV testing among men who have sex with men--National HIV behavioral surveillance system, 20 U.S. metropolitan statistical areas, 2008 and 2011. PLoS One.

[10] Hergenrather KC, Emmanuel D, Durant S, Rhodes SD (2016). Enhancing HIV prevention among young men who have sex with men: a systematic review of hiv behavioral interventions for young gay and bisexual men. AIDS Educ Prev.

[11] Seth P, Walker T, Hollis N, Figueroa A, Belcher L, Centers for Disease Control and Prevention (CDC) (2015). HIV testing and service delivery among Blacks or African Americans--61 health department jurisdictions, United States, 2013. MMWR Morb Mortal Wkly Rep.

[12] Scott HM, Pollack L, Rebchook GM, Huebner DM, Peterson J, Kegeles SM (2014). Peer social support is associated with recent HIV testing among young black men who have sex with men. AIDS Behav.

[13] Washington TA, D'Anna L, Meyer-Adams N, Malotte CK (2015). From their voices: barriers to HIV testing among black men who have sex with men remain. Healthcare (Basel).

[14] Frye V, Wilton L, Hirshfied S, Chiasson MA, Usher D, Lucy D, McCrossin J, Greene E, Koblin B, All About Me Study Team (2015). “Just Because It's Out There, People Aren't Going to Use It.” HIV self-testing among young, black MSM, and transgender women. AIDS Patient Care STDS.

[15] Levy ME, Wilton L, Phillips G, Glick SN, Kuo I, Brewer RA, Elliott A, Watson C, Magnus M (2014). Understanding structural barriers to accessing HIV testing and prevention services among black men who have sex with men (BMSM) in the United States. AIDS Behav.

[16] St Lawrence JS, Kelly JA, Dickson-Gomez J, Owczarzak J, Amirkhanian YA, Sitzler C (2015). Attitudes toward HIV voluntary counseling and testing (VCT) among African American men who have sex with men: concerns underlying reluctance to test. AIDS Educ Prev.

[17] Lauby JL, Milnamow M (2009). Where MSM have their first HIV test: differences by race, income, and sexual identity. Am J Mens Health.

[18] Lippman SA, Moran L, Sevelius J, Castillo LS, Ventura A, Treves-Kagan S, Buchbinder S (2016). Acceptability and feasibility of HIV self-testing among transgender women in San Francisco: a mixed methods pilot study. AIDS Behav.

[19] Oraquick.

[20] Stephenson R, Sullivan PS, Salazar LF, Gratzer B, Allen S, Seelbach E (2011). Attitudes towards couples-based HIV testing among MSM in three US cities. AIDS Behav.

[21] Wagenaar BH, Christiansen-Lindquist L, Khosropour C, Salazar LF, Benbow N, Prachand N, Sineath RC, Stephenson R, Sullivan PS (2012). Willingness of US men who have sex with men (MSM) to participate in couples HIV voluntary counseling and testing (CVCT). PLoS One.

[22] Center for Disease Control and Prevention (2012). 06-Jul-.

[23] Sullivan PS, Salazar L, Buchbinder S, Sanchez TH (2009). Estimating the proportion of HIV transmissions from main sex partners among men who have sex with men in five US cities. AIDS.

[24] Goodreau SM, Carnegie NB, Vittinghoff E, Lama JR, Sanchez J, Grinsztejn B, Koblin BA, Mayer KH, Buchbinder SP (2012). What drives the US and Peruvian HIV epidemics in men who have sex with men (MSM)?. PLoS One.

[25] Ybarra ML, Prescott TL, Philips GL, Bull SS, Parsons JT, Mustanski B (2016). Iteratively developing an mHealth HIV prevention program for sexual minority adolescent men. AIDS Behav.

[26] Mustanski B, Greene GJ, Ryan D, Whitton SW (2015). Feasibility, acceptability, and initial efficacy of an online sexual health promotion program for LGBT youth: the Queer Sex Ed intervention. J Sex Res.

[27] Hightow-Weidman LB, Pike E, Fowler B, Matthews DM, Kibe J, McCoy R, Adimora AA (2012). HealthMpowerment.org: feasibility and acceptability of delivering an internet intervention to young black men who have sex with men. AIDS Care.

[28] Hightow-Weidman LB, Muessig KE, Pike EC, LeGrand S, Baltierra N, Rucker AJ, Wilson P (2015). HealthMpowerment.org: building community through a mobile-optimized, online health promotion intervention. Health Educ Behav.

[29] Dowshen N, Lee S, Matty LB, Castillo M, Mollen C (2015). IknowUshould2: feasibility of a youth-driven social media campaign to promote STI and HIV testing among adolescents in Philadelphia. AIDS Behav.

[30] Bauermeister JA, Pingel ES, Jadwin-Cakmak L, Harper GW, Horvath K, Weiss G, Dittus P (2015). Acceptability and preliminary efficacy of a tailored online HIV/STI testing intervention for young men who have sex with men: the Get Connected! program. AIDS Behav.

[31] Mustanski B, Madkins K, Greene GJ, Parsons JT, Johnson BA, Sullivan P, Bass M, Abel R (2017). Internet-based HIV prevention with at-home sexually transmitted infection testing for young men having sex with men: study protocol of a randomized controlled trial of keep it up! 2.0. JMIR Res Protoc.

[32] Bourne C, Knight V, Guy R, Wand H, Lu H, McNulty A (2011). Short message service reminder intervention doubles sexually transmitted infection/HIV re-testing rates among men who have sex with men. Sex Transm Infect.

[33] Rhodes SD, Vissman AT, Stowers J, Miller C, McCoy TP, Hergenrather KC, Wilkin AM, Reece M, Bachmann LH, Ore A, Ross MW, Hendrix E, Eng E (2011). A CBPR partnership increases HIV testing among men who have sex with men (MSM): outcome findings from a pilot test of the CyBER/testing internet intervention. Health Educ Behav.

[34] Bandura A (1989). Human agency in social cognitive theory. Am Psychol.

[35] Bandura A (1977). Social Learning Theory.

[36] Azjen I, Van Lange PA, Kruglanski AW, Higgens ET (2011). The theory of planned behavior. Handbook of Theories of Social Psychology, Volume 1.

[37] Goffman E (1986). Stigma: Notes of the Management of Spoiled Identity.

[38] Abrams D, Hogg M (1990). Social Identity Theory: Constructive and Critical Advances.

[39] Koblin BA, Mayer KH, Eshleman SH, Wang L, Mannheimer S, del RC, Shoptaw S, Magnus M, Buchbinder S, Wilton L, Liu T, Cummings V, Piwowar-Manning E, Fields SD, Griffith S, Elharrar V, Wheeler D, HPTN 061 Protocol Team (2013). Correlates of HIV acquisition in a cohort of black men who have sex with men in the United States: HIV prevention trials network (HPTN) 061. PLoS One.

[40] Mayer KH, Wang L, Koblin B, Mannheimer S, Magnus M, del Rio C, Buchbinder S, Wilton L, Cummings V, Watson CC, Piwowar-Manning E, Gaydos C, Eshleman SH, Clarke W, Liu T, Mao C, Griffith S, Wheeler D, HPTN061 PT (2014). Concomitant socioeconomic, behavioral, and biological factors associated with the disproportionate HIV infection burden among Black men who have sex with men in 6 U.S. cities. PLoS One.

[41] Mannheimer SB, Wang L, Wilton L, Van TH, del Rio C, Buchbinder S, Fields S, Glick S, Connor MB, Cummings V, Eshleman SH, Koblin B, Mayer KH (2014). Infrequent HIV testing and late HIV diagnosis are common among a cohort of black men who have sex with men in 6 US cities. J Acquir Immune Defic Syndr.

[42] Smylie L, Clarke B, Doherty M, Gahagan J, Numer M, Otis J, Smith G, McKay A, Soon C (2013). The development and validation of sexual health indicators of Canadians aged 16-24 years. Public Health Rep.

[43] Sanchez T, Finlayson T, Drake A, Behel S, Cribbin M, Dinenno E, Hall T, Kramer S, Lansky A, Centers for Disease ControlPrevention (CDC) (2006). Human immunodeficiency virus (HIV) risk, prevention, and testing behaviors--United States, National HIV Behavioral Surveillance System: men who have sex with men, November 2003-April 2005. MMWR Surveill Summ.

[44] Hou S, Luh W (2007). The structure of a web-based HIV testing belief inventory (wHITBI) for college students: the evidence of construct validation. Med Inform Internet Med.

[45] Flowers P, Knussen C, Church S (2003). Psychosocial factors associated with HIV testing amongst Scottish gay men. Psychol Health.

[46] Mashburn AJ, Peterson JL, Bakeman R, Miller RL, Clark LF (2003). Influences on HIV testing among young African-American men who have sex with men and the moderating effect of the geographic setting. J Community Psychol.

[47] Teitcher JE, Bockting WO, Bauermeister JA, Hoefer CJ, Miner MH, Klitzman RL (2015). Detecting, preventing, and responding to “fraudsters” in internet research: ethics and tradeoffs. J Law Med Ethics.

[48] Hosmer D, Lemeshow S (2000). Applied Logistic Regression, 2nd edition.

[49] Sharma A, Stephenson RB, White D, Sullivan PS (2014). Acceptability and intended usage preferences for six HIV testing options among internet-using men who have sex with men. Springerplus.

[50] Golub SA, Gamarel KE (2013). The impact of anticipated HIV stigma on delays in HIV testing behaviors: findings from a community-based sample of men who have sex with men and transgender women in New York City. AIDS Patient Care STDS.

[51] Starks TJ, Rendina HJ, Breslow AS, Parsons JT, Golub SA (2013). The psychological cost of anticipating HIV stigma for HIV-negative gay and bisexual men. AIDS Behav.

[52] Veinot TC, Caldwell E, Loveluck J, Arnold MP, Bauermeister J (2016). HIV testing behavior and social network characteristics and functions among young men who have sex with men (YMSM) in metropolitan Detroit. AIDS Behav.

[53] Garcia J, Parker C, Parker RG, Wilson PA, Philbin M, Hirsch JS (2016). Psychosocial implications of homophobia and HIV stigma in social support networks: insights for high-impact HIV prevention among black men who have sex with men. Health Educ Behav.

[54] Magnus M, Kuo I, Phillips G, Shelley K, Rawls A, Montanez L, Peterson J, West-Ojo T, Hader S, Greenberg AE (2010). Elevated HIV prevalence despite lower rates of sexual risk behaviors among black men in the District of Columbia who have sex with men. AIDS Patient Care STDS.

[55] Millett GA, Peterson JL, Flores SA, Hart TA, Jeffries WL, Wilson PA, Rourke SB, Heilig CM, Elford J, Fenton KA, Remis RS (2012). Comparisons of disparities and risks of HIV infection in black and other men who have sex with men in Canada, UK, and USA: a meta-analysis. Lancet.

[56] Irvin R, Wilton L, Scott H, Beauchamp G, Wang L, Betancourt J, Lubensky M, Wallace J, Buchbinder S (2014). A study of perceived racial discrimination in black men who have sex with men (MSM) and its association with healthcare utilization and HIV testing. AIDS Behav.

[57] Myers J (2015). HIV Self-testing in New York City.

[58] Brewer RA, Magnus M, Kuo I, Wang L, Liu T, Mayer KH (2014). The high prevalence of incarceration history among black men who have sex with men in the United States: associations and implications. Am J Public Health.

[59] Nelson LE, Wilton L, Moineddin R, Zhang N, Siddiqi A, Sa T, Harawa N, Regan R, Dyer TP, Watson CC, Koblin B, Del Rio C, Buchbinder S, Wheeler DP, Mayer KH, HPTN 061 Study Team (2016). Economic, legal, and social hardships associated with HIV risk among black men who have sex with men in six US cities. J Urban Health.

[60] Sullivan PS, Grey JA, Simon Rosser BR (2013). Emerging technologies for HIV prevention for MSM: what we have learned, and ways forward. J Acquir Immune Defic Syndr.

[61] Konstan J, Rosser B, Ross M, Stanton J, Edwards W (2005). The story of subject naught: a cautionary but optimistic tale of internet survey research. J Comput Mediat Commun.

[62] Bauermeister J, Pingel E, Zimmerman M, Couper M, Carballo-Diéguez A, Strecher VJ (2012). Data Quality in web-based HIV/AIDS research: handling invalid and suspicious data. Field methods.

[63] Grey JA, Konstan J, Iantaffi A, Wilkerson JM, Galos D, Simon Rosser BR (2015). An updated protocol to detect invalid entries in an online survey of men who have sex with men (MSM): how do valid and invalid submissions compare?. AIDS Behav.

