# Supplementary material for: Informing the Development of a Mobile Phone HIV Testing Intervention: Intentions to Use Specific HIV Testing Approaches Among Young Black Transgender Women and Men Who Have Sex With Men
Source: JMIR Public Health Surveill. 2017 Jul 7;3(3):e45. doi: 10.2196/publichealth.7397 (PMC5522583; doi:10.2196/publichealth.7397)
Supplement: Multimedia Appendix 1 [file publichealth_v3i3e45_app1.pdf]

Recruitment Sites included: BGC Live, Craig's List, Facebook, Mused Magazine, Scruff, Grindr, local Community organization websites

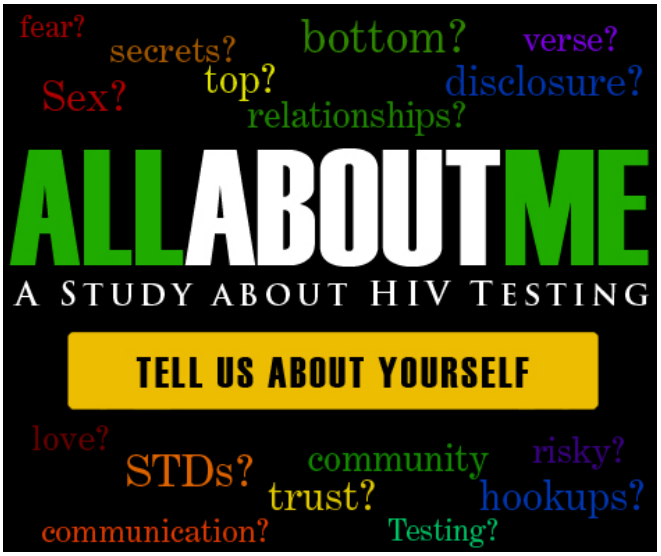

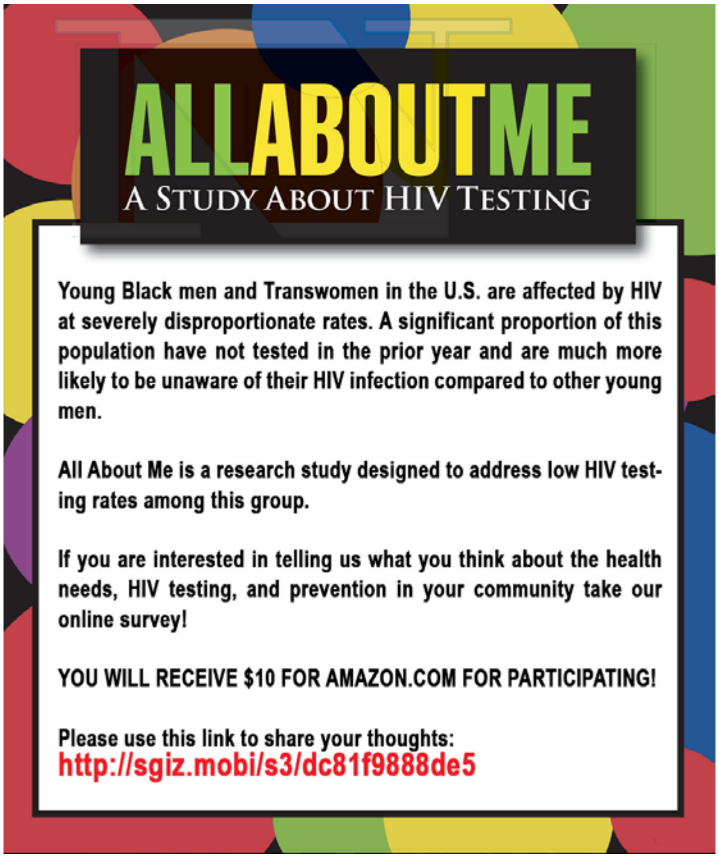

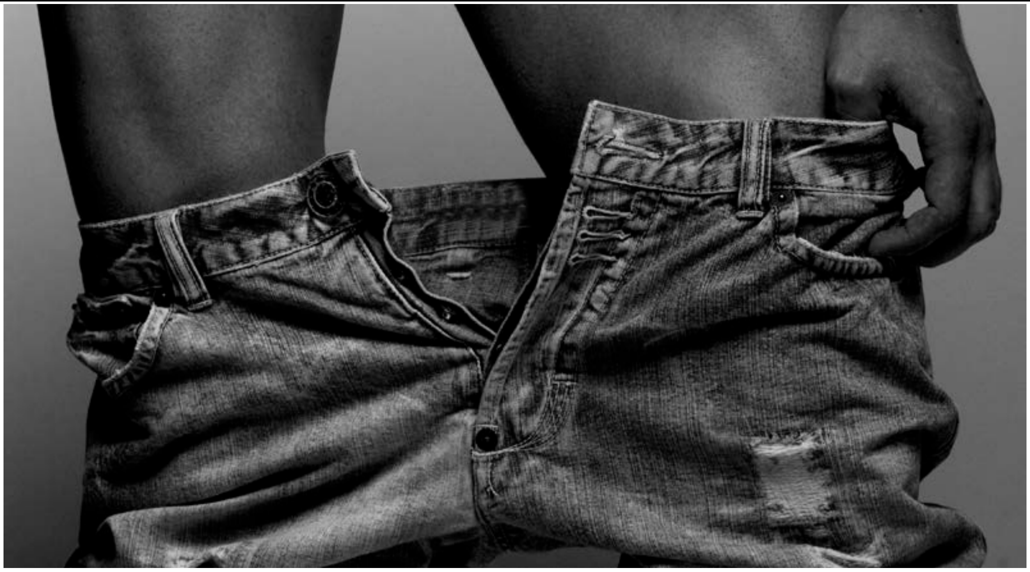

http://sgiz.mobi/s3/479a88ed9cdf Help us learn more about HIV testing in the Black community.
